# Validated Method for the Determination of Piroxicam by Capillary Zone Electrophoresis and Its Application to Tablets

**DOI:** 10.1155/2014/352698

**Published:** 2014-09-09

**Authors:** Arın Gül Dal, Zeynep Oktayer, Dilek Doğrukol-Ak

**Affiliations:** Department of Analytical Chemistry, Faculty of Pharmacy, Anadolu University, 26470 Eskişehir, Turkey

## Abstract

Simple and rapid capillary zone electrophoretic method was developed and validated in this study for the determination of piroxicam in tablets. The separation of piroxicam was conducted in a fused-silica capillary by using 10 mM borate buffer (pH 9.0) containing 10% (v/v) methanol as background electrolyte. The optimum conditions determined were 25 kV for separation voltage and 1 s for injection time. Analysis was carried out with UV detection at 204 nm. Naproxen sodium was used as an internal standard. The method was linear over the range of 0.23–28.79 *µ*g/mL. The accuracy and precision were found to be satisfied within the acceptable limits (<2%). The LOD and LOQ were found to be 0.07 and 0.19 *µ*g/mL, respectively. The method described here was applied to tablet dosage forms and the content of a tablet was found in the limits of USP-24 suggestions. To compare the results of capillary electrophoretic method, UV spectrophotometric method was developed and the difference between two methods was found to be insignificant. The capillary zone electrophoretic method developed in this study is rapid, simple, and suitable for routine analysis of piroxicam in pharmaceutical tablets.

## 1. Introduction

**Figure 1:**
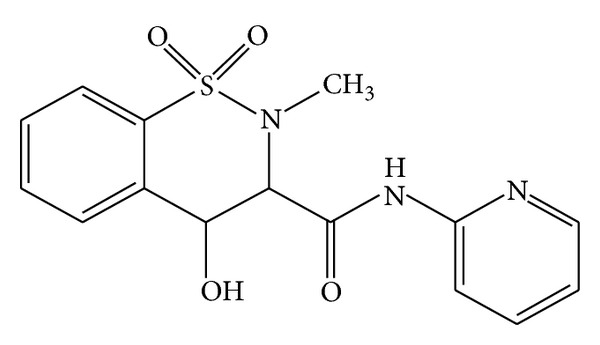
The chemical structure of PIR.

Piroxicam (PIR) (4-hydroxy-2-methyl-N-(pyridine-2-yl)-2H-1,2-benzothiazine-3-carboxamide-1,2-dioxide) is a well-known nonsteroidal anti-inflammatory and analgesic drug, indicated for acute or long-term treatment of inflammation associated with musculoskeletal and joint disorders, such as osteoarthritis, rheumatoid arthritis, and ankylosing spondylitis [1]. The chemical structure is presented in Figure 1.

Nonsteroidal anti-inflammatory drugs (NSAIDs) are the group of analgesics and anti-inflammatory drugs most often used around the world, mainly to treat pain, inflammation, and fever in human. These pharmaceuticals are weak acidic compounds because of their carboxylic groups or keto-enol tautomeric structure with their pK values between 3 and 7. Most of the NSAIDs are chiral, but they are often administered as racemates [2].

Several analytical methods have been described for the determination of PIR, including spectrophotometry [3–9], spectrofluorimetry [10–12], thin-layer chromatography (TLC) [13, 14], liquid chromatography (LC) [9, 13–23], and capillary electrophoresis (CE) [7, 13, 24–27]. CE has emerged as a powerful analytical technique in the analysis of pharmaceutical compounds, such as NSAIDs [1]. The CE methods developed for the determination of PIR in the previous studies were presented without validation except the study of Bartsch et al. [13] which indicated only precision data. Donato et al. [27] reported also only linearity, precision, and accuracy in injectable formulation of PIR and no further validation was shown.

Two capillary electrophoretic methods were reported for pharmaceutical analysis of PIR. One of them was a micellar electrokinetic chromatography (MEKC) method [7] for tablet analysis. There is no information about analysis and migration time for PIR. Linearity range was not very well specified for the determination of PIR; only the quality control (QC) standards compared to pharmaceutical tablets were also presented without validation. Another method reported by Chen and Wu [24] was a capillary zone electrophoretic (CZE) method for the simultaneous determination of seven drugs such as PIR, sulindac, ketoprofen, indomethacin, nimesulide, ibuprofen, and naproxen. Linearity range was 13.24–165 *µ*g/mL and LOD was 3.31 *µ*g/mL with 13 min of migration time. Bartsch et al. [13] demonstrated PIR photodecomposition using three different concentrations (40 *µ*g/mL, 250 *µ*g/mL, and 2 mg/mL) by three methods including high performance LC (HPLC), high performance TLC (HPTLC), and CE. The quantification was evaluated only by external calibration without use of internal standard (IS) and only precision data was presented. Migration time for PIR was reported below 3 min. Boone et al. [25] simultaneously separated six acidic drugs such as hydrochlorothiazide, PIR, ibuprofen, phenobarbital, salicylic acid, and chlorothiazide in spiked serum and urine extracts by using CZE, MEKC, and nonaqueous CE (NACE) techniques as well as basic drugs. This study reports only separation of analytes by CZE with 90 mM sodium tetraborate (pH 8.4), by MEKC with 20 mM sodium phosphate and 50 mM SDS (pH 7.5), and by NACE with 20 mM ammonium acetate in methanol/acetonitrile (9 : 1, v/v) containing 1% acetic acid. PIR (1 *µ*g/mL) eluted up to 4 min in serum and urine by CZE, up to 12 min with ibuprofen as unresolved peaks by MEKC and NACE application were not reported for PIR. The results were also presented without validation. Fillet et al. [26] separated 11 NSAIDs including PIR in a background electrolyte solution of 100% methanol and 13 NSAIDs in a second background electrolyte solution of acetate in methanol and acetonitrile (70 : 30, v/v) by using nonaqueous system showing PIR resolution from other drugs and its migration time appeared up to 8 min in both systems but the reported study did not cover any method validation or pharmaceutical application.

Therefore there is a need for the determination of PIR in pharmaceutical tablets by CZE with full validation. The aim of this study is to develop a validated, simple, and rapid CZE method for the analysis of PIR in pharmaceutical tablets. The proposed method was linear over wide range of 0.23–28.79 *µ*g/mL and was validated in relation to precision, accuracy, selectivity, and sensitivity (with LOD of 0.07 *µ*g/mL for PIR) in QC standards and tablet matrix. Method accuracy was also confirmed by using UV spectrophotometric method.

## 2. Experimental

### 2.1. Chemicals

PIR and naproxen sodium (NAP) as IS were purchased from Sigma Chemical Co. (USA). All other chemicals were of analytical grade and were purchased from Merck GmbH Company (Germany). Ultrapure water was purified with a Milli-Q system of Millipore (USA). Commercial PIR tablets (Felden Flash, Pfizer, Turkey) containing 20 mg PIR were obtained from a local pharmacy.

### 2.2. Apparatus

CE (Thermo Separation Products, Spectra Phoresis 100, USA) was performed with SPD-10A model UV detector (Shimadzu, Japan) and data was processed by CR-7A model (Shimadzu, Japan) integrator. Compounds were separated in 75 *µ*m i.d. fused-silica tubing of 53.6 cm effective and 68.2 cm total length (Agilent Technologies, USA).

Spectrophotometric studies were conducted using UV-2401 model spectrophotometer (Shimadzu, Japan). The pH of the solutions was measured by a model of M-822 pH meter (Electro-mag, Turkey). All solutions were sonicated in a B-220 model ultrasonic bath (Branson, USA) before injection.

### 2.3. Preparation of Solutions

Standard PIR and NAP solutions were prepared in methanol and distilled water, respectively. The final concentration of the IS was always 6.76 *µ*g/mL.

Background electrolyte (BGE) consisted of 10 mM borate buffer containing 10% (v/v) methanol adjusted to pH 9.0.

For UV spectrophotometric experiments 0.36, 0.72, 1.08, and 1.44 *µ*g/mL PIR solutions were prepared in methanol and methanol was used as blank. These amounts of PIR were added to unknown tablet solution and absorbance was recorded.

### 2.4. CZE Procedure

Fused-silica capillary used for the first time was conditioned by flushing with 1.0 M NaOH for 30 min followed by 0.1 M NaOH, ultrapure water, and BGE for 10 min, respectively.

Each day, the capillary was washed and conditioned by rinsing for 10 min with each of 0.1 M NaOH, ultrapure water, and BGE, respectively. The samples were then injected into the fused-silica capillary filled with BGE, by vacuum injection for 1 s. Between each run the capillary was rinsed with 0.1 M NaOH (2 min), distilled water (2 min), and BGE (2 min). At the end of each working day, it was washed with 0.1 M NaOH and ultrapure water for 10 min and left with aspirated air. During analysis the applied potential was +25 kV, under voltage-controlled conditions. Detection was performed at 204 nm.

### 2.5. Validation Studies

The method was validated according to ICH guidelines for validation of analytical procedures [28].

The precision of the method was determined by the measurement of repeatability (intraday) and intermediate precision (interday). Standard solution of PIR 1.79 *µ*g/mL was injected on three consecutive days, six times in a day.

The linearity of the method was investigated with 8 concentrations in the range of 0.23–28.79 *µ*g/mL. Linearity was evaluated by linear regression analysis using the least square regression method. Calibration plots were chosen in this range and six concentrations were injected for three consecutive days.

0.23 *µ*g/mL, 1.79 *µ*g/mL, and 14.41 *µ*g/mL PIR solutions were used for accuracy studies. The accuracy in matrix was determined by adding PIR onto matrix to give the final concentrations of 0.26, 2.56, and 25.58 *µ*g/mL. The matrix was prepared as common tablet excipients such as hydroxypropyl methylcellulose (7%), lactose monohydrate (60%), magnesium stearate (1%), polyethylene glycol 4000 (5%), povidone (5%), maize starch (5%), talc (1%), and titanium dioxide (1%).

Quantification was accomplished on the basis of PIR to NAP normalized peak area ratios (rPN) [i.e., (peak area of PIR/migration time of PIR)/(peak area of NAP/migration time of NAP)].

### 2.6. Tablet Analysis

The method was applied to a PIR tablet as in the pharmacopeial rules [29].

For the application of the method, 10 Felden Flash (each containing 20 mg PIR) tablets were weighed to calculate the average weight of a tablet. 10 tablets were then powdered and the average amount of a tablet was weighed. It was dissolved in 25 mL methanol and sonicated for 10 min. The solution was centrifuged for 10 min at 5000 rpm. The supernatant was diluted with methanol to the appropriate concentration to measure the amount of PIR.

## 3. Results and Discussion

The pKa value of PIR is 6.3. Buffer pH has an influence on the degree of ionization of the molecules, their electrophoretic mobilities, and electroosmotic flow. Therefore, alkaline conditions were considered to be suitable for the determination of PIR according to its molecular structure.

### 3.1. Optimization of the Method

Experiments were performed to determine the optimum conditions.

Borate buffer was selected for BGE to provide the solubility of PIR. Initially, borate buffer was investigated in the concentration range of 10–25 mM and 15 mM borate provided the optimum results.

Methanol was preferred for the modifier of the BGE because it decreases the mobility of the components in capillary column. It was found out that better migration times and good peak shapes were obtained with the use of 10% (v/v) methanol.

The effect of the pH was tested in the range of 8.5 and 9.5. Well-shaped peaks appeared with the use of pH 9.0. At this pH, the electroosmotic flow has reached a maximum, fairly constant mobility and PIR is completely ionized, so that its corresponding electrophoretic mobility was not affected by any slight variations in pH. PIR was in an anionic form in the BGE of pH 9.0 and eluted after electroosmosis.

Well-shaped peaks of PIR appeared at +25 kV and the applied potential was kept at +25 kV. Despite higher voltages which are preferred in CE, voltages higher than 25 kV caused problems such as current generation, poor separation, and resolution between PIR and IS.

An injection time of 1 s was used because longer duration caused zone broadening.

**Figure 2:**
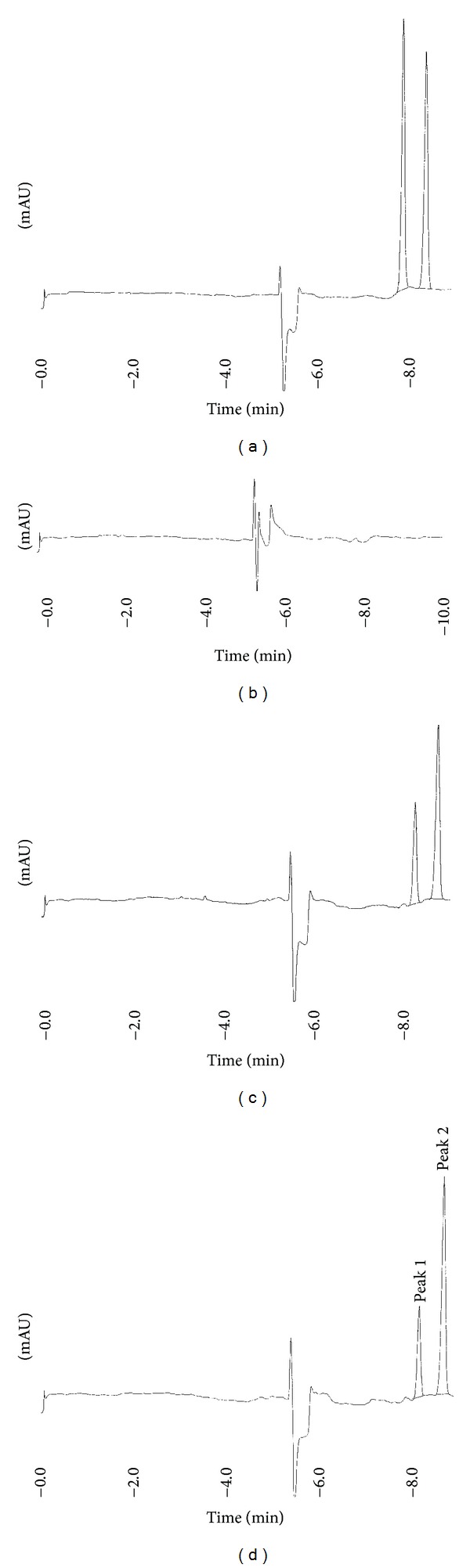
The electropherogram of (a) standard PIR (7.20 *µ*g/mL) and IS (6.76 *µ*g/mL), (b) matrix solution, (c) PIR spiked (2.58 *µ*g/mL) matrix solution (IS 6.76 *µ*g/mL), and (d) tablet solution containing PIR (2.58 *µ*g/mL) (IS 6.76 *µ*g/mL). Peak 1 is piroxicam (PIR); peak 2 is naproxen sodium (NAP) as internal standard (IS).

The optimum conditions determined were as follows: 10 mM borate buffer at pH 9.0, containing 10% (v/v) methanol for BGE. +25 kV applied potential and 1 s of injection time were used as the instrumental parameters. Then NAP was injected as IS, and it appeared in appropriate time and resolution in the electropherogram. Under these analytical and instrumental conditions, the migration times were 8.11 ± 0.03 min for PIR and 8.60 ± 0.04 min for NAP (*n* = 6) as seen in Figure 2(a).

Electroosmotic mobility was calculated as 6.61 × 10^−6^ cm^2^/s*·*V. Methanol was used as a neutral marker. Electrophoretic mobilities were calculated as −1.5 × 10^−4^ cm^2^/s*·*V for PIR and −1.66 × 10^−4^ cm^2^/s*·*V for NAP. The electrophoretic mobility for PIR was given as −22.8 ×  10^−5^ cm^2^/s*·*V in only one of the CE studies which used nonaqueous system [26] and it is not comparable with this study.

### 3.2. Validation of the Method

The precision of the method was examined as individual days (intraday) and intermediate precision (interday) of rPN, expressed as a RSD% of a series of measurements. The results are demonstrated in Table 1.

Statistical evaluation of the precision results showed that RSD% values both intraday and interday were below 2%. These results indicate that method precision is analytically acceptable [28].

The linearity of the method was examined in the range of 0.23–28.79 *µ*g/mL and the calibration plots were chosen in this range. The statistical data evaluated by using rPN were presented in Table 2.

Good correlation fit the equation as *y* = 52511*x* − 0.0027 with correlation coefficient of 0.9999.

Accuracy was tested in both PIR and matrix solutions. The percentage error was calculated by use of the formula [(found concentration − spiked concentration)/spiked concentration] × 100%. Results are shown in Table 3.

The acceptance criteria are not higher than 2% deviation from the nominal value for accuracy [28]. The percent recoveries were found almost 100% for drug substance and drug product, and accuracy was much less than the acceptable criteria. Therefore, the accuracy results are highly satisfactory for the determination of PIR.

LOD and LOQ were estimated as [(standard deviation of regression equation)/(slope of the regression equation)] by multiplying with 3.3 and 10, respectively. The LOD was 0.07 *µ*g/mL and the LOQ was 0.19 *µ*g/mL.

Specificity was performed using tablet inactive ingredients to assure that these common tablet dosage form ingredients could interfere with the peaks of interest. The data indicated that these ingredients did not interfere with PIR and IS peaks indicating the specificity of this method as seen in Figures 2(b) and 2(c).

### 3.3. Application of the Method to Determination of PIR in Tablets

The method was applied to pharmaceutical tablets (Felden tablet) containing 20 mg active material. Samples were prepared as described in the experimental section. The peaks of tablet samples carried the characteristics of standard PIR, and there was no interference originating from the matrix as seen in Figure 2(d). The values of RSD% were below 2% and the percent recoveries were almost 100% as seen in Table 4. The content of a tablet is in the limits of USP-24 requirements [29].

For the determination of PIR in commercial tablets, to compare the results of CZE method, UV spectrophotometric method was developed. The progressed CZE method was compared to a UV spectrophotometric method. The study was carried out at 204 nm. Analysis was performed with standard addition method to show the selectivity parameter. A good linear relationship fitting the equation [*y* = 84691*x* + 0.3974], with high correlation coefficient (0.9999), was obtained between added amounts to tablet and absorbance values.

The UV spectrophotometric experimental values obtained for PIR are presented in Table 4. The results showed that UV spectrophotometric method was accurate and precise. And also the common tablet dosage form ingredients did not interfere with PIR peaks shown by standard addition technique, so the method was selective for PIR. UV spectrophotometry and CZE, used for the determination of PIR in tablets, were statistically compared with* t-* and* F* tests. They are demonstrated in Table 4.

The calculated* t* value for the methods was 1.60 and was less than the table* t* value (1.73) at 95% confidence level. Calculated* F* values were 0.77 and 2.14 for CZE and UV spectrophotometry, respectively. At 95% confidence level, the ratio of two* F* values was calculated as 2.77 and was less than the table* F* value (5.05). The difference between the two methods was found to be insignificant as a result of* t-* and* F* tests.

Although some of the chromatographic methods [14–20, 22, 23] seemed to have lower LODs and shorter analysis times, CZE methods need small amount of analyte volumes, and it takes shorter time to condition the capillary. This study has lower sensitivity than a previous chromatographic one [21]. Among the CE studies the sensitivity of this method either is better than in [7, 24, 27] or could not be compared because the sensitivity is not declared [13, 25, 26]. Some of the CE studies [13, 26, 27] have shorter analysis time than this study. But also the developed method here has shorter analysis time with better resolution than a CE study [24] and could not be compared with some of them [7, 25] because the analysis time is not declared.

According to the best of our knowledge, the specificity of the proposed method was not shown in the tablet matrix in previous CE studies and the method sensitivity is the best one. Therefore, it could be a promising method for the possible application of PIR in biological matrix because of low detection limit.

## 4. Conclusion

In this study, a simple and rapid capillary electrophoretic method for the determination of PIR in tablets was developed and validated. Validation of the method has been shown with parameters of linearity, precision, LOD and LOQ, accuracy, and specificity. It was found that all the results of percent coefficient of variation are below 2% showing the method was valid. The CZE method developed here is proposed for the routine analysis of PIR in pharmaceutical formulations.

## Figures and Tables

**Table 1:** Precision data for 1.79 *μ*g/mL PIR.

	Intraday			Interdays
	Day 1 (*n* = 6)	Day 2 (*n* = 6)	Day 3 (*n* = 6)	Whole days (*n* = 18)
Mean	0.284	0.286	0.286	0.285
SD	0.004	0.004	0.004	0.005
RSD%	1.581	1.494	1.476	1.827
CI (*P* < 0.05)	±0.004	±0.003	±0.003	±0.002

SD is standard deviation, RSD is relative standard deviation, CI is confidence interval, and *P* < 0.05 is probability level of 95% for *n* = 6 experiments (intraday) and for *n* = 18 experiments (interdays).

**Table 2:** Calibration data for PIR.

	Intraday			Interdays
	Day 1 (*n* = 6)	Day 2 (*n* = 6)	Day 3 (*n* = 6)	Whole days (*n* = 18)
*a*	51518	52811	51240	51857
*b*	0.005	−0.002	0.007	0.003
*R*	0.9999	0.9999	0.9999	0.9998
±Sr	0.020	0.020	0.015	0.059
RSD% of *a*	1.089	1.070	0.857	1.832
CI (*P* < 0.05)	±463	±466	±362	±394

*a* is slope, *b* is intercept, *R* is correlation coefficient, Sr is standard deviation of regression equation, RSD is relative standard deviation, CI is confidence interval, and *P* < 0.05 is probability level of 95% for *n* = 6 experiments (intraday) and for *n* = 18 experiments (interdays).

**Table 3:** Accuracy data for PIR.

Standard	Day 1 (*n* = 3)	Added (*μ*g/mL)	0.23	1.79	14.41
		Found (mean ± SD)	0.23 ± 0.003	1.8 ± 0.02	14.54 ± 0.11
		Recovery%	102.61	100.26	101.15
		SE%	2.61	0.26	1.15
		RSD%	1.44	1.12	0.76
	Day 2 (*n* = 3)	Added (*μ*g/mL)	0.23	1.79	14.41
		Found	0.23 ± 0.003	1.79 ± 0.002	14.51 ± 0.06
		Recovery%	99.95	99.76	100.78
		SE%	−0.05	−0.23	0.78
		RSD%	1.49	1.12	0.41
	Day 3 (*n* = 3)	Added (*μ*g/mL)	0.23	1.79	14.41
		Found	0.23 ± 0.001	1.8 ± 0.01	14.61 ± 0.1
		Recovery%	102.45	100.47	101.61
		SE%	2.45	0.47	1.61
		RSD%	0.68	0.63	0.81
	Whole days (*n* = 9)	Added (*μ*g/mL)	0.23	1.79	14.41
		Found	0.23 ± 0.003	1.8 ± 0.01	14.54 ± 0.1
		Recovery%	101.67	100.16	101.18
		SE%	1.67	0.16	1.18
		RSD%	1.67	0.72	0.69

Matrix	(*n* = 3)	Added (*μ*g/mL)	0.26	2.56	25.58
		Found	0.25 ± 0.03	2.56 ± 0.01	25.8 ± 0.1
		Recovery%	99.76	100.21	100.94
		SE%	−0.24	0.21	0.94
		RSD%	1.26	0.64	0.65

SD is standard deviation, SE is standard error, and RSD is relative standard deviation for *n* = 3 experiments (standard and matrix) and for *n* = 9 experiments (standard, whole days).

**Table 4:** Application and comparison of the methods.

	CE (*n* = 3)			UV spectrophotometry (*n* = 5)			
Added PIR (*μ*g/mL)	0.26	2.58	25.8	0.36	0.72	1.08	1.44
Recovery%	101.78	102.62	102.72	101.74	99.88	100.43	101.10
SD	1.185	0.847	0.395	1.78	1.57	0.94	1.11
RSD%	1.165	0.826	0.385	1.75	1.57	0.94	1.10
CI∗	±1.99	±1.43	±0.66	±1.69	±1.49	±0.89	±1.06

T*_calculated_	1.60						
T*_table_	1.73						

F*_calculated_	2.77						
F*_table_	5.05						

CE is capillary electrophoresis, UV is ultraviolet, PIR is piroxicam, SD is standard deviation, RSD is relative standard deviation, CI is confidence interval, ∗ is probability level of 95%, *t* is Student's *t*-test, and *F* is *F*-test for *n* = 3 (CE) and for *n* = 5 (UV) experiments.
